# DiffFSRE: Diffusion-Enhanced Prototypical Network for Few-Shot Relation Extraction

**DOI:** 10.3390/e26050352

**Published:** 2024-04-23

**Authors:** Yang Chen, Bowen Shi

**Affiliations:** 1 State Key Lab of Software Development Environment, Beihang University, Beijing 100191, China; yangchen_nlsde@buaa.edu.cn; 2 School of Journalism, Communication University of China, Beijing 100024, China

**Keywords:** relation extraction, diffusion model, prototypical networks, entropy, few-shot learning

## Abstract

Supervised learning methods excel in traditional relation extraction tasks. However, the quality and scale of the training data heavily influence their performance. Few-shot relation extraction is gradually becoming a research hotspot whose objective is to learn and extract semantic relationships between entities with only a limited number of annotated samples. In recent years, numerous studies have employed prototypical networks for few-shot relation extraction. However, these methods often suffer from overfitting of the relation classes, making it challenging to generalize effectively to new relationships. Therefore, this paper seeks to utilize a diffusion model for data augmentation to address the overfitting issue of prototypical networks. We propose a diffusion model-enhanced prototypical network framework. Specifically, we design and train a controllable conditional relation generation diffusion model on the relation extraction dataset, which can generate the corresponding instance representation according to the relation description. Building upon the trained diffusion model, we further present a pseudo-sample-enhanced prototypical network, which is able to provide more accurate representations for prototype classes, thereby alleviating overfitting and better generalizing to unseen relation classes. Additionally, we introduce a pseudo-sample-aware attention mechanism to enhance the model’s adaptability to pseudo-sample data through a cross-entropy loss, further improving the model’s performance. A series of experiments are conducted to prove our method’s effectiveness. The results indicate that our proposed approach significantly outperforms existing methods, particularly in low-resource one-shot environments. Further ablation analyses underscore the necessity of each module in the model. As far as we know, this is the first research to employ a diffusion model for enhancing the prototypical network through data augmentation in few-shot relation extraction.

## 1. Introduction

Relation extraction [1] is a foundational task in information extraction, focusing on discerning the semantic relationships between the head and tail entities within contexts. Traditional supervised learning methods [2,3] excel in relation extraction tasks. However, data quality and scale play a major role in their performance. In practice, manually annotating high-quality data is a labor-intensive or time-consuming task, leading to the susceptibility of these supervised models to data scarcity and making it challenging for them to generalize effectively. To address the challenge of constructing large-scale datasets, Mintz et al. [4] proposed a novel distant supervision (DS) mechanism. This approach automatically labels training instances by aligning existing knowledge graphs (KGs) with text. Experience suggests that DS can automatically label a sufficient number of training instances, but these data typically cover a limited number of relationships in real-world scenarios. Many relationships are long-tail, resulting in insufficient data. Current DS models overlook the issue of long-tail relationships, making them struggle to extract comprehensive information from pure text.

Observations from real-world scenarios indicate that humans often learn new knowledge after several iterations. Therefore, few-shot learning methods [5,6,7] have become a focal point in recent research. Few-shot learning initially achieved success in the computer vision (CV) community [8,9] and was recently introduced to relation extraction by Han et al. [10], proposing the few-shot relation extraction task (FSRE). In recent years, various methods [11,12] have been proposed to address the challenges of few-shot relation extraction. It is typical for these methods to be initially trained on large volumes of data for existing relation types and then rapidly adapted to smaller amounts of data for new relation types. One popular algorithmic framework for few-shot learning is meta-learning [13,14]. This method samples from external data containing disjoint relation sets to construct multiple sets of few-shot learning tasks. The model is then optimized to learn cross-task knowledge, enabling it to quickly adapt to new tasks. A straightforward and efficient meta-learning algorithm is the prototypical network [15]. It aims at learning an appropriate metric space where query instances are categorized based on their distance from class prototypes. Despite achieving significant success in the few-shot relation extraction domain, methods [15,16] based on prototypical networks often suffer from overfitting relational classes in the training set, resulting in a mediocre generalizing capability to unseen relations. Consequently, the challenge of overcoming the inherent limitations of data scarcity remains a substantial hurdle for the academic community.

On the other hand, diffusion models [17,18,19] have demonstrated remarkable performance in image generation, gaining widespread attention in the field of artificial intelligence. Researchers have also applied these models to the field of natural language processing (NLP) and have begun exploring their generative capabilities in this domain [20,21]. To date, diffusion models have been extensively utilized in generative NLP tasks, including unconditional text generation, controllable text generation, machine translation, and text simplification. Moreover, recent studies indicate that diffusion models maintain impressive generative performance in low-data scenarios [22,23].

Inspired by the aforementioned research, we explore leveraging diffusion models for data augmentation. Combining the diffusion model with a prototypical network to address the overfitting issues in few-shot relation extraction, we propose a diffusion model-enhanced prototypical network framework. Initially, we design and train a conditional relation generation diffusion model on the training dataset. Given descriptions of relation classes, the diffusion model can generate pseudo-sample features for data augmentation. Building upon the trained diffusion model, we further present a pseudo-sample-enhanced prototypical network. This augmentation is able to provide more accurate representations for prototype classes in the prototypical network, thereby alleviating overfitting and better generalizing to unseen relation classes. Additionally, in order to enhance the adaptability of the prototypical network to pseudo-sample data, we introduce a pseudo-sample-aware attention mechanism through a cross-entropy loss, further improving the model’s performance. To validate the proposed framework, we conduct a comprehensive set of experiments and analyses. According to experimental results, our proposed method is superior to existing approaches.

We outline the principal contributions as follows:

- We design and train a controllable conditional relation generation diffusion model on the relation extraction dataset, which can generate the corresponding instance representation according to the relation type description.
- We propose a prototypical network framework enhanced by a diffusion model, enabling data augmentation through the generation of pseudo-sample data. This augmentation is able to provide more accurate representations for prototype classes in the prototypical network, thereby alleviating overfitting and better generalizing to unseen relation classes. Additionally, we introduce a pseudo-sample-aware attention mechanism to boost the adaptability of the prototypical network to pseudo-sample data through a cross-entropy loss, further improving the model’s performance. As far as we know, this is the first research that employs a diffusion model to enhance the prototypical network through data augmentation in few-shot relation extraction.
- In order to validate the proposed method, we conduct extensive experiments. The results indicate that our proposed approach significantly outperforms existing methods, particularly in low-resource-shot environments. Further ablation analyses underscore the necessity of each module in the model.

## 2. Related Work

Few-shot relation extraction is a crucial research area whose goal is to learn and extract semantic relationships between entities with only a limited number of annotated samples. The majority of current research utilizes prototypical networks [15] for few-shot relation extraction (FSRE), intending to acquire an appropriate prototypical vector for each relation. Gao et al. [16] proposed a model called HATT-Proto, building on previous research. This model combines convolutional neural network encoding and prototypical networks, introducing an innovative hybrid attention mechanism at both instance and feature levels. These two attention mechanisms are employed to reduce interference from noisy samples and emphasize crucial features, thereby enhancing the model’s performance in few-shot relation extraction tasks. Fan et al. [24] adopted a more fine-grained embedding encoding approach. They utilized convolutional neural networks for encoding both sentences and phrases, introduced auxiliary loss functions, and enhanced the prototypical network by large-margin learning. This innovation strengthened the model’s generalization ability in identifying tail relations, further improving the accuracy of few-shot relation extraction. Ye et al. [25] presented a multilevel matching and aggregation network. This approach not only retained previous encoding methods but also interactively encoded query set instances and class prototypes. This novel method achieved state-of-the-art performance at that time. Wen et al. [12] innovatively proposed a few-shot relation extraction model that successfully integrates the Transformer [26] architecture with the prototypical network. By leveraging the multi-head attention mechanism, the model achieved significant improvement in feature extraction, thereby enhancing the accuracy of relation extraction. Ding et al. [27] made innovative improvements based on MTB [28]. They proposed an effective method to directly learn relation representations from unstructured text, considering the perspective of prototype metrics. This approach optimized the measurement between sentences and abstracted the core characteristics of relation classes by inferring prototypes, thereby further enhancing the performance of relation extraction. Liu et al. [29] introduced a straightforward yet powerful approach incorporating relation information into the prototypical network. The fundamental concept involves incorporating relation representations through a direct addition operation rather than designing intricate structures.

By integrating rich entity and relation information from knowledge graphs, researchers can effectively improve the accuracy of few-shot relation extraction, enabling models to maintain high accuracy and generalization even in the presence of limited samples. This interdisciplinary information fusion strategy has brought new breakthroughs and possibilities to the few-shot relation extraction task. Yu et al. [30] introduced prior knowledge from knowledge graphs to enrich prototype learning. They not only utilized this knowledge to enhance the model’s generalization capability but also innovatively introduced a prototype regularization mechanism to consider the similarity between different prototypes. Yang et al. [31] proposed a few-shot relation extraction model named ConceptFERE, which incorporates entity concept enhancement. In this model, they adopted pre-trained concept embeddings proposed by Shalaby et al. [32] to represent entity concept information. This embedding method not only allows the model to gain a deeper understanding of entity concepts but also enhances its generalization capability in few-shot relation extraction tasks. He et al. [33] introduced a virtual prompt pre-training approach involving the projection of the virtual prompt into the latent space, which was followed by fusion with parameters of the pre-trained language model.

## 3. Preliminary Study

This section provides the preliminary knowledge necessary to understand our approach, including task formulation, the prototypical network and diffusion models.

### 3.1. Task Formulation

**Figure 1:**
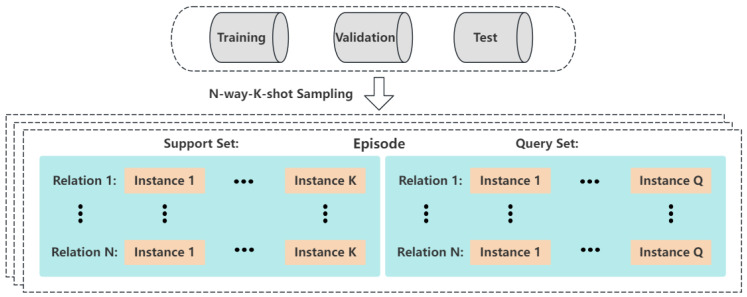
The depiction of sampling N-way-K-shot episodes.

Typically, research on few-shot relation extraction (FSRE) is carried out under the N-way-K-shot configuration where models undergo training and testing across a set of episodes with each episode randomly generated from distinct training and test datasets. Essentially, the relation classes used for testing are not present in the training dataset. As illustrated in Figure 1, episodes are randomly selected from the training, validation or test datasets. Each episode comprises N relation classes, and every class is divided into K instances (forming the support dataset S) and multiple instances (forming the query dataset Q). In each episode, every instance (s,e,y) consists of a given sentence s, two marked entities $e=(e_{1},e_{2})$, and the corresponding relation label y, where $e_{1}$ and $e_{2}$ denote the head and tail entities, respectively. Few-shot relation extraction aims to identify all relationships in the query set Q of all episodes.

### 3.2. Prototypical Network

The prototypical network holds significant significance in the current research on few-shot relation extraction. Its fundamental research question revolves around how to learn class prototypes effectively to better represent a specific class. Our approach is built upon the prototypical network proposed by Snell et al. [15]. Specifically, the prototypical network model employs a non-parametric classifier mapping query points to the class prototypes nearest to them in the learned embedding space. For a class *c* in the predefined class set, its prototype $z^{c}$ is computed by the following formula:

(1) $$z^{c}=\frac{1}{K}\sum_{i}f\left(x^{c,i}\right)$$

where $f\left(x^{c,i}\right)$ outputs the embedding vector of the sample $x^{c,i}$ in the support set. For any query sample $x^{q}$, the predicted distribution is computed using a softmax of its distances from all classes’ prototypes in the embedding space:

(2) $$p\left(y_{n}^{q}=c|x^{q}\right)=\frac{\exp(-d\left(f\left(x^{q}\right),z^{c}\right))}{\sum_{c^{\prime }}\exp\left(-d\left(f\left(x^{q}\right),z^{c^{\prime }}\right)\right)}$$

where d(·,·) represents the Euclidean distance function.

### 3.3. Diffusion Model

Before delving into our framework, let us provide a concise overview of fundamental concepts essential for comprehending diffusion models (DDPMs) [17,18]. Firstly, a forward noising process is assumed in diffusion models, which gradually adds noise to real data $x_{0}$. The forward noising process, denoted as $q(x_{t}|x_{t-1})$, can be described as a Markov process that starts with a sample $x_{0}∼q\left(x_{0}\right)$ and introduces Gaussian noise at each timestep t. In summary, the forward noising process can be formalized as follows:

(3) $$\begin{array}{c} q\left(x_{T}|x_{0}\right):=\prod_{t=1}^{T}q\left(x_{t}|x_{t-1}\right) \end{array}$$

(4) $$\begin{array}{c} q\left(x_{t}|x_{t-1}\right):=N\left(x_{t};\sqrt{1-\beta_{t}}x_{t-1},\beta_{t}I\right) \end{array}$$

where N represents the Gaussian distribution, and ${\left\{\beta \right\}}_{t=0}^{T}$ is a set of hyperparameters controlling the magnitude of the noise. By setting $\alpha_{t}:=1-\beta_{t}$ and $\bar{\alpha_{t}}:=\prod_{s=1}^{t}\alpha_{s}$, $x_{t}$ can be expressed in terms of $x_{0}$:

(5) $$\begin{array}{c} x_{t}=\sqrt{{\bar{\alpha }}_{t}}x_{0}+\sqrt{1-{\bar{\alpha }}_{t}}\epsilon \end{array}$$

(6) $$\begin{array}{c} \epsilon ∼N(0,I).\; \end{array}$$

When we set $\alpha_{T}$ to tend sufficiently toward 0, $q\left(x_{T}|x_{0}\right)$ approximates a standard Gaussian distribution.

The reverse denoising process allows us to reconstruct samples from pure Gaussian noise and is typically approximated as a Markov chain. We can use a neural network $p_{\theta }$ to estimate it:

(7) $$\begin{array}{cc} \; & p_{\theta }\left(x_{0:T}\right)=p\left(x_{T}\right)\prod_{t=1}^{T}p_{\theta }\left(x_{t-1}|x_{t}\right)\\ \; & p_{\theta }\left(x_{t-1}|x_{t}\right)∼N\left(\mu_{\theta }\left(x_{t},t\right),\Sigma_{\theta }\left(x_{t},t\right)\right) \end{array}$$

where

(8) $$p_{\theta }\left(x_{t-1}|x_{t}\right):=N\left(x_{t-1};\mu_{\theta }\left(x_{t},t\right),\sigma_{t}^{2}I\right),\;\mu_{\theta }\left(x_{t},t\right):=\frac{1}{{\sqrt{\alpha }}_{t}}\left(x_{t}-\frac{1-\alpha_{t}}{\sqrt{1-{\bar{\alpha }}_{t}}}\epsilon_{\theta }\left(x_{t},t\right)\right)$$

The above $\epsilon_{\theta }\left(x_{t},t\right)$ is fitted by a neural network model with the optimization objective being

(9) $$L_{\theta }=E_{t,x_{0},\epsilon }\left[{∥\epsilon -\epsilon_{\theta }\left(\sqrt{{\bar{\alpha }}_{t}}x_{0}+\sqrt{1-{\bar{\alpha }}_{t}}\epsilon ,t\right)∥}^{2}\right].$$

Once the model is trained, during the generation process, we can sample according to the following formula:

(10) $$x_{t-1}=\mu_{\theta }\left(x_{t},t\right)+\sigma_{t}\epsilon =\frac{1}{\sqrt{\alpha_{t}}}\left(x_{t}-\frac{1-\alpha_{t}}{\sqrt{1-{\bar{\alpha }}_{t}}}\epsilon_{\theta }\left(x_{t},t\right)\right)+\sigma_{t}\epsilon$$

## 4. Methodology

This subsection provides a detailed description of our proposed framework, encompassing the conditional relation generation diffusion model and the prototypical network enhanced by generative data augmentation.

### 4.1. Conditional Relation Generation Diffusion Model

**Relational feature encoding of samples**: For each sample instance (x,e,y) in the support set *S*, we obtain its relational feature encoding by utilizing the pre-trained language model BERT [34]. Specifically, we start by adding special tokens [E1] and [E2] before the head and tail entities in the example sentence x. Subsequently, we input it into the pre-trained BERT language model, from which we can extract contextual vectors for [E1] and [E2] from its final output layer. Finally, we concatenate these vectors with the encoded sentence vector, resulting in the relational feature encoding H:

(11) $$H=concat(h_{x},h_{\left[E1\right]},h_{\left[E2\right]})$$

where $h_{x}$ represents the output vector at the [CLS] position of the sample sentence, and $h_{\left[E1\right]}$ and $h_{\left[E2\right]}$ represent the output vectors of BERT at the positions of [E1] and [E2], respectively.

**The forward process**: For each sample in the dataset, based on the previous subsection, we obtain its relational feature vector. Our diffusion model considers the relational feature vector as the initial sample feature $x_{0}$. Subsequently, we undergo a forward noising process, and after T timesteps, we progressively generate the noise sample sequence $x_{0},x_{1},\cdots ,x_{T}$; the process is analogous to DDPM [18].

**The reverse process**: Our conditional diffusion model takes relation descriptions as the extra input. In this setting, the reverse process becomes $p(x_{t-1}|x_{t},r)$. Following the classifier-free guidance diffusion model [35], the DDPM sampling process can be directed to sample *x* with a high probability p(x|r) by

(12) $${\hat{\epsilon }}_{\theta }\left(x_{t},r\right)=\epsilon_{\theta }\left(x_{t},r\right)+s\;\cdot \;\nabla_{x}\log p\left(r|x\right)\propto \epsilon_{\theta }\left(x_{t},r\right)+s\;\cdot \;\left(\epsilon_{\theta }\left(x_{t},r\right)-\epsilon_{\theta }\left(x_{t},\emptyset \right)\right)$$

where s>1 represents the scale of the guidance (note that s = 1 corresponds to standard sampling). The unconditional diffusion model is impletemented by randomly discarding r in training while substituting it with a learnable “NA” embedding.

**Figure 2:**
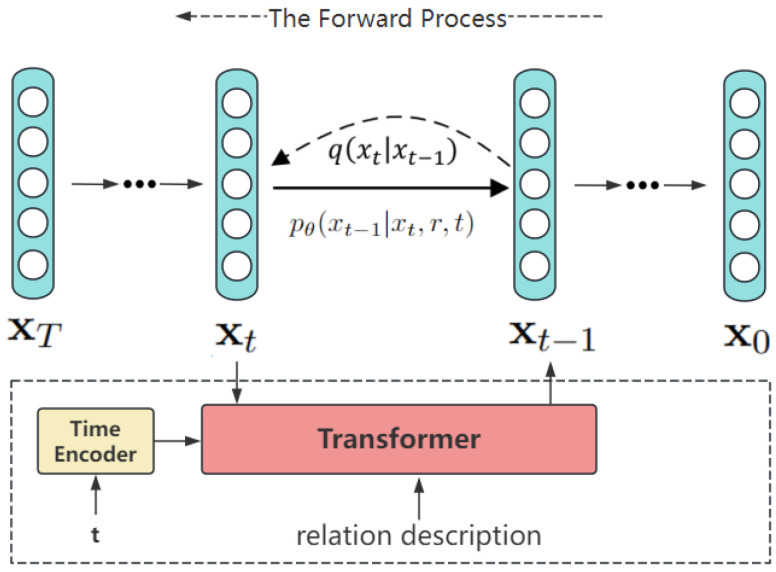
The illustration of conditional relation generation diffusion model.

**Denoising network**: Our reverse denoising process employs a neural network $f_{\theta }\left(x_{t},r,t\right)$ to fit. At each timestep *t*, it takes three parameters: $x_{t}$ as the current noisy sample encoding, *r* as the relation description, and *t* as the current timestep. Specifically, we use a bidirectional Pre-LN transformer [26] with 12 layers and a hidden dimension of d = 768. To incorporate information about the timestep, we follow established practices commonly used in image diffusion. Specifically, following the approach of Vaswani et al. [26], we represent the timestep t using sinusoidal positional encoding. This encoding is then processed through a Multi-Layer Perceptron (MLP) to obtain a time embedding. We add the time embedding to the embedding of the relation description input sequence. Then, we feed them into the encoder of the transformer model. The embedding of noise sample $x_{t}$ in current timestep t is fed into the decoder of the transformer model, which yields the embedding of noise $\epsilon_{\theta }^{t}$ in the decode process. Finally, $x_{t-1}$ can be obtained through the sampling formula. We illustrate the whole process in Figure 2.

### 4.2. Enhanced Prototypical Network with Pseudo-Sample Augmentation

This subsection outlines how to utilize the well-trained conditional relation generation diffusion model to enhance the prototypical network model, thereby improving its performance in few-shot scenarios. Prototypical network methods require a small number of samples for each class to compute embeddings for prototype classes. This can lead to overfitting and a lack of generalization to new classes not seen during training. To address this issue, we use the conditional relation generation diffusion model to generate a certain quantity of pseudo-sample features for each relation class, mitigating the problem of overfitting.

**Pseudo-sample relational feature generation**: In the training process, for each episode in the N-ways-K-shots setting, we generate $N_{g}$ pseudo-samples for each relation class. The hyperparameter $N_{g}$ can vary with different values of K in practice. The generative pseudo-samples are added to the support set, which is used for training the episode loss computation in the next process. The training detail is illustrated in Algorithm 1.

During the validation or testing process, since the training data for the conditional relation generation diffusion model do not include the relation classes in the validation and test set, we conduct a fine-tuning process on the validation and test set. This additional fine-tuning ensures that the diffusion model possesses the capability to generate pseudo-samples for the new relation classes. Specifically, in the N-ways-K-shots setting, our diffusion model is fine-tuned in each episode to align to the episodic process. Subsequently, we generate multiple pseudo-sample features for each relation class within the support set, providing additional data support for the subsequent prototypical network.

**Pseudo-Sample-Aware Attention Mechanism**: Due to the considerable noise present in the pseudo-data generated by the diffusion model, it is essential for the model to assign distinct weights to real and pseudo-sample features. Hence, we devise a prototypical network with a pseudo-sample-aware attention mechanism. Specifically, in the N-way-K-shot setup, where each class in the support set S has K instances, we initially generate $n_{k}$ pseudo-samples for each class. For any instance q in the query set Q, we calculate the prototype class representation ${\hat{h}}_{r}$ for every class in the support dataset S concerning instance q. It can be formalized as shown below:

(13) $$\begin{array}{c} {\hat{h}}_{r}=\sum_{j=0}^{L_{r}-1}a_{j}^{r}\;\cdot \;f\left(h_{j}^{r}\right) \end{array}$$

(14) $$\begin{array}{c} a_{j}^{r}=\frac{\exp(g\left(f\left(h_{q}\right)\right)\;\cdot \;g\left(f\left(h_{r}^{j}\right)\right))}{\sum_{i=0}^{L_{r}-1}\exp\left(g\left(f\left(h_{q}\right)\right)\;\cdot \;g\left(f\left(h_{r}^{i}\right)\right)\right)}, \end{array}$$

(15) $$\begin{array}{c} g\left(h\right)=\left\{\begin{array}{cc} Linear_{q}\left(h\right) & \;if\;instance\;of\;h\;\in Q\\ Linear_{g}\left(h\right) & \;if\;instance\;of\;h\;\in G\\ Linear_{r}\left(h\right) & \;if\;instance\;of\;h\;\in S \end{array}\right. \end{array}$$

where $h_{q}$ represents the sample feature encoding for instance q in the query set, and $h_{r}^{j}$ signifies the feature encoding for the j-th sample of relation r within the support dataset. G denotes the pseudo-sample set generated by the diffusion model. The function f(·) is a Multi-Layer Perceptron (MLP) utilized to map the original sample features to a metric space, while g(·) is a piecewise linear function with the design of its linear layers tailored to different sample types. Finally, the cross-entropy loss for an episode can be formulated as shown below:

(16) $$L=\frac{1}{N_{C}N_{Q}}\sum_{x\in Q}(d\left(f\left(x\right),c_{k}^{x}\right)+log\sum_{k^{\prime }}\left(-d\left(f\left(x\right),c_{k^{\prime }}^{x}\right)\right)$$

where $c_{k}^{x}$ represents the embedding of the k-th relation prototype for query instance x, $N_{C}$ denotes the number of relation classes in the episode, and $N_{Q}$ represents the number of instances for every class in the query set.
**Algorithm 1:** Training episode loss computation for our prototypical networks.
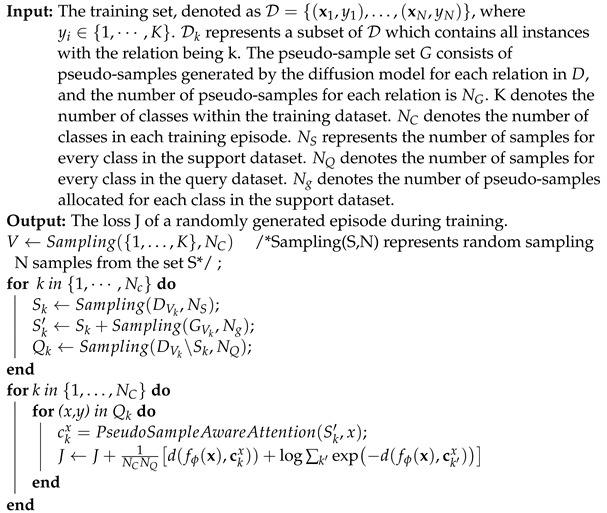


## 5. Experiments

### 5.1. Datasets

Our model undergoes evaluation on two widely used datasets for few-shot relation extraction: FewRel 1.0 [10] and FewRel 2.0 [36]. FewRel 1.0 is an extensive FSRE dataset which is human-annotated and constructed based on articles from Wikipedia. It encompasses 100 relations with 700 instances per relation. There are 64 relations in the training dataset, 16 relations in the validation dataset, and 20 relations in the testing dataset. FewRel 2.0 uses the same training set as FewRel 1.0. However, the testing dataset in FewRel 2.0 is constructed from the biomedical domain, ensuring non-overlap with any relation in the training dataset. This test set comprises 25 relations with each relation consisting of 100 instances.

### 5.2. Baselines

We choose recent competitive methods as our baselines for comparison with our approach. These methods primarily include the following:

**Proto-BERT** [15]: A prototype network model based on BERT, utilizing BERT as the encoding layer and employing the traditional prototype network algorithm.

**TD-Proto** [37]: An enhanced prototype network utilizing relation and entity descriptions. This approach incorporates a collaborative attention module to extract beneficial information and guiding cues from both sentences and entities. A gating mechanism is introduced to dynamically fuse these two types of information, resulting in an instance with knowledge awareness.

**MLMAN** [25]: A multilevel matching and aggregation prototype network. Through this approach, query instances and each support set are encoded interactively by taking into account both local and instance-level matching information. By aggregating its supporting instances, the ultimate class prototype of each support set is calculated with weights derived based on the query instance.

**CP** [38]: An entity-masked contrastive pre-training framework. They initially construct a large-scale dataset from Wikidata, comprising 744 relations and 867,278 sentences. Subsequently, they proceed to pre-train the current BERT model on the obtained dataset and finally fine-tune the dataset using a prototype network, achieving high extraction accuracy on the FewRel 1.0.

**HCRP** [39]: An enhanced Proto-BERT which introduces a hybrid prototype learning method, producing informative prototypes to capture subtle interrelation variations. A task adaptive focal loss is also proposed to prioritize challenging tasks during training.

**SimpleFSRE** [29]: A prototype network model enhanced by relation descriptions.

**GM_GEN** [40]: A model generation framework consisting of a universal model for all tasks and numerous task-specific small models to deal with separate tasks.

**LPD** [41]: A label prompt dropout approach that efficiently utilizes the relation description.

### 5.3. Implementation Details

Our conditional relation generation diffusion model follows the training and sampling procedure of the classifier-free diffusion model [35]. The hyperparameter $p_{uncond}$ is set to 0.2. The training timestep is configured as 1000. We follow the DDIM [42] to accelerate sampling and set the sampling step to 10. We utilize AdamW [43] as the optimizer. The experiments are deployed on 8 Tesla V100 32 GB GPUs. The classification accuracy of our models is calculated by averaging over 1000 randomly sampled episodes from the validation and test sets.

### 5.4. Experimental Results

The experimental results on the FewRel 1.0 validation and testing sets are presented in Table 1. Compared with existing prototype network-based methods, our DiffFSRE model significantly outperforms them. It is important that our method performs notably better than the comparison models across all N-way-K-shot configurations. Additionally, it is worth noting that our model exhibits a larger performance improvement in the more challenging 1-shot setting compared to the 5-shot setting. These results indicate that our model demonstrates strong generalization capabilities, better addressing the data scarcity issue in demanding few-shot scenarios. Similar conclusions can be drawn from the results on the FewRel 2.0 dataset, as shown in Table 2.

### 5.5. Ablation Study

We conduct thorough ablation studies via systematically disabling various elements of our model to understand their distinct influences. The comparison of our model with its variants variants is presented in Table 3.

**Effect of Conditional Relation Generation Diffusion Model**: To validate the effectiveness of our proposed conditional relation generation diffusion model, we conducted corresponding ablation experiments. In Table 3, ‘*w/o* diffusion’ indicates that we do not use the diffusion model to generate pseudo-samples. Instead, we randomly select a certain number of samples from the support set as pseudo-samples and include them in the model training. It can be observed from the table that compared to the model with randomly selected pseudo-samples, the diffusion model-generated pseudo-samples lead to a significant improvement in model performance, demonstrating the effectiveness of the conditional relation generation diffusion model.

**Effect of Pseudo-Sample-Aware Attention Mechanism**: To investigate the role of the pseudo-sample-aware attention mechanism, we conducted corresponding ablation experiments. In Table 3, ‘*w/o* attention’ indicates that we removed the pseudo-sample-aware attention mechanism during training and used the computation method of the original prototype network. It can be observed that not using the pseudo-sample-aware attention mechanism resulted in a significant decline in model performance. This suggests that for the prototype network, the ability to distinguish between generated pseudo-samples and real samples is crucial, thereby confirming the effectiveness of our proposed pseudo-sample-aware attention mechanism.

### 5.6. Analysis of the Number of Pseudo-Samples

**Figure 3:**
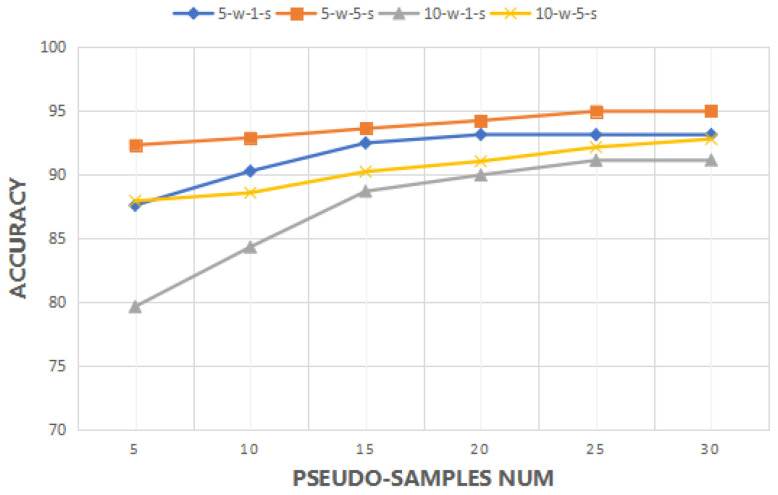
The model’s performance under different settings on FewRel 1.0, the accuracy on the validation dataset is reported.

To investigate the impact of generating different amounts of pseudo-samples on our prototype network framework, we conducted multiple comparative experiments. Specifically, we trained our DiffFSRE model with varying numbers of pseudo-samples in four scenarios: “5-way-1-shot, 5-way-5-shot, 10-way-1-shot, and 10-way-5-shot”. The experimental results are presented in Figure 3. We can observe that as the number of generated pseudo-samples increases in all four scenarios, the model’s performance steadily improves. This indicates that enhancing the prototype network model with pseudo-samples is effective in mitigating the overfitting issues of the prototype model.

## 6. Conclusions

In this study, we explore to utilize a diffusion model for data augmentation to address the overfitting issue of prototypical networks. We propose a diffusion model-enhanced prototypical network framework. Specifically, we design and train a controllable conditional relation generation diffusion model on the relation extraction dataset, which can generate the corresponding instance representation according to the relation description. Building upon the trained diffusion model, we further present a pseudo-sample-enhanced prototypical network, which is able to provide more accurate representations for prototype classes, thereby alleviating overfitting and better generalizing to unseen relation classes. Additionally, we introduce a pseudo-sample-aware attention mechanism to enhance the model’s adaptability to pseudo-sample data through a cross-entropy loss, further improving the model’s performance. A series of experiments are conducted to prove our method’s effectiveness. The results indicate that our proposed approach significantly outperforms existing methods, particularly in low-resource one-shot environments. Further ablation analyses underscore the necessity of each module in the model. To our knowledge, this is the first research that uses a diffusion model to enhance the prototypical network through data augmentation in few-shot relation extraction.

## Figures and Tables

**Table 1:** Validation set/test set accuracy for different models on FewRel 1.0. The best results are shown in bold.

Model	5-Way-1-Shot	5-Way-5-Shot	10-Way-1-Shot	10-Way-5-Shot
**Proto-HATT** [16]	72.65/74.52	86.15/88.40	60.13 / 62.38	76.20/80.45
**MLMAN** [25]	75.01/—	87.09/90.12	62.48/—	77.50 / 83.05
**MTB** [28]	—/91.10	—/95.40	—/84.30	—/91.80
**Proto-BERT** [15]	82.92/80.68	91.32/89.60	73.24/71.48	83.68/82.89
**TD-Proto** [37]	—/84.76	—/92.38	—/74.32	—/85.92
**CP** [38]	88.29/90.85	92.77/95.60	80.50/83.89	88.61/90.61
**HCRP** [39]	90.90/93.76	93.22/95.66	84.11/89.95	87.79/92.10
**LPD** [41]	88.84/93.79	90.65/95.07	79.61/89.39	82.15/91.08
**SimpleFSRE** [29]	91.29/94.42	94.05/96.37	86.09/90.73	89.68/93.47
**GM_GEN** [40]	92.65/94.89	95.62/96.96	86.81/91.23	91.27/94.30
**DiffFSRE**(ours)	**93.14**/**96.35**	**94.97**/**97.86**	**91.12**/**95.64**	**92.79**/**96.47**

**Table 2:** Accuracy of various competitive models on FewRel 2.0 test set. Results of contrast models are from previous papers.

Model	5-Way-1-Shot	5-Way-5-Shot	10-Way-1-Shot	10-Way-5-Shot
Proto-BERT	40.12	51.50	26.45	36.93
HCRP	76.34	83.03	63.77	72.94
LPD	77.82	86.90	66.06	78.43
GM_GEN	76.67	91.28	64.19	84.84
**DiffFSRE** (ours)	89.41	92.72	83.96	85.52

**Table 3:** Ablation experiments on the FewRel 1.0; the accuracy on the validation set is reported.

Model	5-Way-1-Shot	5-Way-5-Shot	10-Way-1-Shot	10-Way-5-Shot
Full model	93.14	94.97	91.12	92.79
*w/o* diffusion	80.17	82.35	72.48	81.39
*w/o* attention	90.24	91.56	89.38	90.21

## Data Availability

The data presented in this study are available on request from the corresponding author.
